# Requirements of health policy and services journals for authors to disclose financial and non-financial conflicts of interest: a cross-sectional study

**DOI:** 10.1186/s12961-017-0244-2

**Published:** 2017-09-19

**Authors:** Assem M. Khamis, Maram B. Hakoum, Lama Bou-Karroum, Joseph R. Habib, Ahmed Ali, Gordon Guyatt, Fadi El-Jardali, Elie A. Akl

**Affiliations:** 1 Faculty of Health Sciences, American University of Beirut, Beirut, Lebanon; 2 Clinical Research Institute, American University of Beirut Medical Center, Beirut, Lebanon; 3 Center for Systematic Reviews for Health Policy and Systems Research (SPARK), American University of Beirut, Beirut, Lebanon; 4 Faculty of Medicine, American University of Beirut, Beirut, Lebanon; 5 Department of Health Research Methods, Evidence, and Impact, McMaster University, Hamilton, ON Canada; 6 Department of Medicine, McMaster University, Hamilton, ON Canada; 7 Department of Internal Medicine, American University of Beirut Medical Center, P.O. Box 11-0236, Riad-El-Solh Beirut, 1107 2020 Beirut, Lebanon

## Abstract

**Background:**

The requirements of the health policy and services journals for authors to report their financial and non-financial conflicts of interest (COI) are unclear. The present article aims to assess the requirements of health policy and services journals for authors to disclose their financial and non-financial COIs.

**Methods:**

This is a cross-sectional study of journals listed by the Web of Science under the category of ‘Health Policy and Services’. We reviewed the ‘Instructions for Authors’ on the journals’ websites and then simulated the submission of a manuscript to obtain any additional relevant information made available during that step. We abstracted data in duplicate and independently using a standardised form.

**Results:**

Out of 72 eligible journals, 67 (93%) had a COI policy. A minority of policies described how the disclosed COIs of authors would impact the editorial process (34%). None of the policies had clear-cut criteria for rejection based on the content of the disclosure. Approximately a fifth of policies (21%) explicitly stated that inaccurate or incomplete disclosures might lead to manuscript rejection or retraction. No policy described whether the journal would verify the accuracy or completeness of authors’ disclosed COIs. Most journals’ policies (93%) required the disclosure of at least one form of financial COI. While the majority asked for specification of source of payment (71%), a minority asked for the amount (18%). Overall, 81% of policies explicitly required disclosure of non-financial COIs.

**Conclusion:**

A majority of health policy and services journal policies required the disclosure of authors’ financial and non-financial COIs, but few required details on disclosed COIs. Health policy journals should provide specific definitions and instructions for disclosing non-financial COIs. A framework providing clear typology and operational definitions of the different types of COIs will facilitate both their disclosure by authors and reviewers and their assessment and management by the editorial team and the readers.

**Electronic supplementary material:**

The online version of this article (doi:10.1186/s12961-017-0244-2) contains supplementary material, which is available to authorized users.

## Background

The Institute of Medicine defines a conflict of interest (COI) as “*a set of circumstances that creates a risk that professional judgment or actions, regarding a primary interest, will be unduly influenced by a secondary interest*” [1]. In healthcare, the primary interest is to assist in the advancement of health research and generate the useful knowledge for patients, while the secondary interest is personal gains, either financial or non-financial [2, 3]. In 1998, the International Committee of Medical Journal Editors (ICMJE) required submitting authors to include a covering letter including “*a statement of financial or other relationships that might lead to a conflict of interests*” [4]. Since then, it appears that journals and health organisations (e.g. professional societies that produce practice guidelines), including organisations whose mandate is to deal with issues of health policy, have developed or amended their policies regarding COIs [5–7].

A number of reports highlight how COIs can affect health policymaking [8–11]. One of these reports relates to the implementation of the Framework Convention on Tobacco Control (FCTC) in China. After signing the convention in 2003 and starting 2009, China printed on-pack warnings according to the FCTC agreement [8]. However, the on-pack warnings did not follow global standards [8]. According to Wan et al. [8], the State Tobacco Monopoly Administration, responsible for implementing the FCTC agreement, had a share in the largest tobacco company in the country, an obvious COI.

India is another example of how COIs may have affected tobacco control health policies. Despite the Indian government signing the FCTC in 2003, tobacco users represented up to 35% of the adults in 2010 according to the Global Adult Tobacco Survey [12]. Rao et al. [9] investigated 100 public documents addressing the competing interests in tobacco control policies in India. They identified COIs at three levels, namely at the individual, organisations/government and policy planning levels. Specifically, stakeholders and advocators holding positions in governments and institutions responsible for setting and implementing tobacco policies shared or had ownership in tobacco companies [9]. In one illustrative example, a former minister and the director of a tobacco company, served as a member of the powerful group of ministers deciding on pictorial warnings on tobacco packs.

Thailand succeeded over a 20 year period in reducing tobacco use among adults [11]. One of the main success factors was assigning the state-owned Thai Tobacco Monopoly to the Ministry of Finance, while the Ministry of Health was responsible for tobacco control [10, 11]. The Ministry of Health assembled a National Committee for the Control of Tobacco Use, whose members must not be part of other tobacco agencies to exclude any potential COI [10, 11].

Evidence-informed health policymaking should be based on a disinterested evaluation of the best available evidence [13]. One study found that health policymakers in the Middle East do refer to evidence in their decision-making processes [14, 15]. Several studies, although not in the policymaking field, found a positive association between authors reporting COIs and an increased likelihood of their studies reporting positive results [16, 17]. This highlights the need for policymakers to assess the potential for bias in the evidence on which they are considering basing their decisions. Therefore, requiring authors to disclose their COIs will allow policymakers to better assess the possible bias introduced by those interests.

We recently conducted a survey of core clinical journals and found that all but one of the 117 journals had a COI policy [18]. Many of those journals were, however, deficient in terms of disclosure of non-financial COIs, disclosure of financial COIs of authors’ family members and institutions, and effects of disclosed COIs on the editorial process. However, there is no similar survey focusing on health policy and health services journals. Therefore, we undertook an assessment of the requirements of health policy and services journals for authors to disclose their financial and non-financial COIs.

## Methods

### Overall design

The study consisted of (1) a review of published documents describing the journals’ COI policy and of (2) the collection of relevant information during a simulated manuscript submission.

**Fig. 1:**
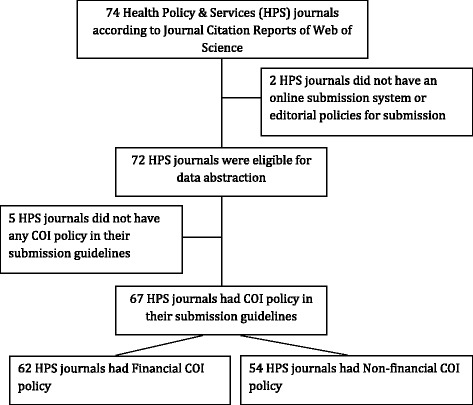
Flow diagram for selection of journals

### Definitions

We used the definition of COI by the Institute of Medicine, as provided above [1]. We defined a COI policy as one that requires, as a minimum, the authors to disclose their COIs.

### Eligibility criteria

Our study population consisted of all journals listed by the Web of Science database under the category of ‘Health Policy and Services’. We excluded journals that do not have an online submission system. Given that the research addressed the journals’ administrative processes related to COI disclosure policies, the Institutional Review Board of the American University of Beirut deemed the study as not human subject research and, as such, was exempted from ethical review.

### Data collection process

We collected information from three different sources, namely (1) instructions and forms accessible on the journal website; (2) instructions and forms accessible from the journal online submission system or sent by email when simulating an article submission; (3) and instructions and forms accessible on the publisher’s website, when redirected there by the journal online submission system.

The simulated submission consisted of the following. First, we logged into the journal online submission system and submitted an empty manuscript under the manuscript category ‘original research’, or its equivalent, on the journal online submission system. We also submitted a cover letter explaining the objective and methods of the current study. We included in the submission one email address for the ‘submitting author’ and another for a ‘co-author’. Then, they examined for any relevant email sent to the author and co-author email addresses.

After conducting calibration exercises, we abstracted data using a standardised and pilot tested data abstraction form and an accompanying instructions document. We abstracted data in a duplicate and independent manner and resolved discrepancies through discussion or through the help of a third reviewer.

### Data collected

#### General characteristics of the journal

- Impact factor according to the Social Science Citation Index using June 2016 version of the Journal Citation Report
- Journal category other than ‘Health Policy and Services’ category (according to the Journal Citation Report Science Edition 2015) (to better characterise the profile of journals included in the study)
- Membership of the ICMJE, according to the ICMJE website (as this could be potentially associated with adoption of certain COI policies)
- Membership of the Committee on Publication Ethics (COPE) (also potentially associated with adoption of certain COI policies)
- Open access status at the time of publication
- Affiliation with a professional organisation
- Publisher
- Requirement for authors to comply with reporting statements (e.g. CONSORT)

#### Characteristics of the COI disclosure policy

- Existence of a COI policy
- Basis of the COI policy (e.g. publisher, ICMJE)
- Form used for COI disclosure
- COI disclosure submission method (e.g. in the cover letter, in the manuscript)
- Relation of COI disclosure to submitted work
- Timing of COI disclosure relative to submission
- Time period for which disclosure of COI is required
- The handling of COI disclosures (verification, reporting in the publication, effect of disclosures and inaccurate or incomplete disclosures on the editorial process)

#### Requirements for financial COI disclosure

- Individuals required to disclose financial COI (e.g. authors, family members, institutions or associated departments)
- Types of financial COI to disclose (e.g. grant, personal fees, indirect financial support, stock ownership, direct employment)
- Specification of the source and/or amount of payment/service

In addition, we collected the types of non-financial COI for which disclosure is required. In the instructions document, we provided guidance to distinguish between the COI disclosures (being specific to the authors) and funding statements (being specific to the study).

### Data analysis

We checked data for any missing or erroneous entries. For the descriptive analyses of journals’ characteristics, we used mean and standard deviation (or median and interquartile range) for continuous variables and frequencies and percentages for categorical variables. We conducted regression analyses to identify relationships between (1) existence of a COI disclosure policy, (2) explicit requirement for disclosure of financial COI, and (3) explicit requirement for disclosure of non-financial COI, and the independent variables of COPE, ICMJE and impact factor.

## Results

Out of the 74 journals listed by the Web of Science under the ‘Health Policy and Services’ category, we excluded two journals that publish papers by invitation only and have no publicly available submission guidelines (Fig. 1).

### General characteristics of the journals

Table 1 shows the general characteristics of 72 included journals. Only seven percent of the journals were members of the ICMJE, while 75% were members of the COPE. The majority of journals were open access at the time of publication (n = 59; 82%) and were affiliated with a professional organisation (63%).

**Table 1:** General characteristics of journals (n = 72)

Variable	n (%)
Impact factor, median (interquartile range)	1.624 (1.024–2.320)
Journal categories other than Health Policy	38 (53)
Public Environmental & Occupational Health	19 (26)
Economics	5 (7)
Other	14 (19)
Membership of the ICMJE	5 (7)
Membership of the COPE	54 (75)
Open Access at the time of publication	59 (82)
By default	11 (19)
By authors choice	48 (81)
Affiliation with a professional organisation	45 (63)
Publisher	
Springer^a^	11 (15)
Wiley-Blackwell	8 (11)
BioMed Central^a^	7 (10)
Sage	7 (10)
Elsevier	5 (7)
Taylor & Francis	5 (7)
Lippincott Williams & Wilkins	4 (6)
Oxford University Press	3 (4)
Cambridge University Press	3 (4)
Other	19 (26)
Requirement for authors to comply with reporting statements^b^	
CONSORT	18 (25)
PRISMA	17 (24)
STROBE	15 (21)
COREQ	11 (15)
None	50 (69)
Existence of a conflicts of interest policy	67 (93)

^a^Springer announced the acquisition of BioMed Central (BMC) Group in October 2008

^b^Journals may have more than one option that applies

*COPE* Committee on Publication Ethics, *ICMJE* International Committee of Medical Journal Editors

### Characteristics of the COI disclosure policies

Out of the 72 included journals, 67 (93%) had a COI policy (Fig. 1). Table 2 shows the characteristics of those policies. Of these policies, 93% explicitly required disclosure of financial and 81% of non-financial COIs. Although most journals’ policies required COI disclosure for the submitted work (87%), only one (2%) required COI disclosure for work other than the submitted work. The latter journal specifically asks authors for having been paid as a consultant (or in a similar capacity) by a company with a vested interest in the product being studied, on issues unrelated to the product being studied. The remaining nine journals (14%) did not specify to what the required disclosure should relate. A quarter of the journals (25%) specified the time period for which disclosure is required; the most frequently specified period was 36 months (64% of the 17).

**Table 2:** Characteristics of the conflicts of interest (COI) disclosure policies (n = 67)

Variable	n (%)
Disclosure policy specifies financial COI	62 (93)
Disclosure policy specifies non-financial COI	54 (81)
Disclosure policy is based on:^a^	
Journal’s policy	54 (81)
Publisher’s policy	32 (48)
Other (e.g. based on ICMJE recommendations)	5 (8)
Form used for COI disclosure^a^	
Narrative statement	59 (88)
ICMJE disclosure form	13 (19)
Modified ICMJE form	13 (19)
Publisher form	11 (16)
Journal form	6 (9)
COI disclosure submission method^a^	
In the cover letter	18 (27)
In the body of the manuscript	42 (63)
Online as part of submission	50 (75)
Via email	2 (3)
COI disclosure required in relation to:^a^	
Submitted work	58 (87)
Work outside the submitted work	1 (2)
Not specified	9 (14)
Timing of COI disclosure relative to submission	
After submission	4 (6)
After revision or acceptance	5 (8)
Specification of time period for which disclosure is required	17 (25)
Time period for which disclosure is required^b^	
Preceding 12 months	1 (6)
Preceding 24 months	3 (18)
Preceding 36 months	11 (64)
Preceding 5 years	2 (12)
Near future	7 (41)
Time period is anchored to:^b^	
Time of submission	14 (82)
Time of initiation of study	2 (12)
Not specified	1 (6)
More detailed COI disclosure may be requested	7 (10)

^a^Journals may have more than one option that applies

^b^n = 17 (i.e. number of journals that specified time period for disclosure)

*ICMJE* International Committee of Medical Journal Editors

### Publication of disclosure and effect on the editorial process

Of the 67 policies, two thirds (67%) explicitly stated that authors’ COI disclosure statements would be published within the manuscript, while only one explicitly stated that they would not. Of the 67 policies, 23 (34%) explicitly described how the disclosed COIs of authors would impact the editorial process. Two policies (9%) reported that COIs would have no effect on the decision to accept or reject the paper. The remaining policies (91%) suggested that the disclosed COIs of authors might affect the decision to accept or reject (Additional file 1). None of the policies had clear-cut criteria for rejection based on the content of the disclosure. Only one policy described how the COIs of the editorial team are managed during the editorial process.

About a fifth of policies (21%) explicitly stated that inaccurate or incomplete disclosures might lead to manuscript rejection or retraction (Additional file 2). No policy described whether the journal would verify the accuracy or completeness of authors’ disclosed COIs.

### Requirements for financial COI disclosure

Sixty-two out of the 67 journals’ policies (93%) required at least one form of financial COI disclosure (Fig. 1). Table 3 shows the requirements for financial COI disclosure for these 62 policies. The policies addressed financial relationships of the authors (100%), their family members (35%) and the authors’ institutions or associated departments (27%). The top three types of financial COI that policies required disclosure for were stock ownership, grants and direct employment. While the majority asked for specification of source of payment (71%), a minority asked for the amount (18%).

**Table 3:** Requirements for financial conflicts of interest (COI) disclosure (n = 62)^a^

Variable	n (%)
Individuals required to disclose financial COI:	
Authors	62 (100)
Family members	22 (35)
Institutions/associated departments	17 (27)
Other^b^	1 (2)
Types of financial COI:	
Stock ownership	51 (82)
Grant	49 (79)
Direct employment	49 (79)
Serving as an advisor, consultant, or public advocate	43 (69)
Personal fees	40 (65)
Disclosure of patents (planned, pending or issued)	40 (65)
Indirect financial support	37 (60)
Honoraria for speaking, writing or reviewing	29 (47)
Royalties	27 (44)
Speaker bureaus or board membership	23 (37)
Other^c^	9 (15)
Not specified	6 (10)
Source of payment	44 (71)
Amount of payment	11 (18)
Irrespective of amount	7 (64)
For amounts above a specific cut-off value^d^	4 (36)

^a^Journals may have more than one option that applies; n = 62 refers to the policies that require the disclosure of at least one type of financial COI

^b^‘Other’ includes medical writers and other contributors

^c^‘Other’ includes mutual fund ownership (n = 4); economic and commercial (n = 1); commercial (n = 1); fiduciary interest (n = 1); sources of funds/earnings (n = 1); any financial relationships (n = 1)

^d^The respective cut-off values for the four journals were US $750.00 per year for three journals and $10,000 for one journal

### Requirements for non-financial COI disclosure

Fifty-four out of the 67 (81%) journals’ policies required at least one form of non-financial COI disclosure (Fig. 1). Table 4 provides the descriptors used to refer to non-financial COI for these 54 policies. The top three descriptors that non-financial COI policies required disclosure for were ‘personal relationship’ (54%), ‘non-financial COI’ (33%) and ‘professional’ (28%).

**Table 4:** Descriptors used to refer to non-financial conflicts of interest (COI) for which disclosure is required (n = 54)^a^

Non-financial COI terms	n (%)
Personal relationship	29 (54)
Other	21 (39)
Non-financial COI	18 (33)
Professional	15 (28)
Academic competition	12 (22)
Personal opinion	10 (19)
Academic	9 (17)
Intellectual passion	9 (17)
Religious views	8 (15)
Political	8 (15)
Personal	8 (15)
Intellectual	7 (13)
Ideological	7 (13)
Competing loyalties	5 (9)
Advocacy groups/institutional advocacy	5 (9)
Institutional	3 (6)
Legal relationship	3 (6)
Governmental	1 (2)

^a^Journals may have more than one option that applies; n = 54 refers to the policies that require the disclosure of at least one type of non-financial COI

There were no significant associations between the journals’ characteristics such as COPE, ICMJE membership and impact factor and (1) the existence of COI disclosure policy, (2) the explicit requirement for disclosure of financial or (3) the explicit requirement for disclosure of non-financial COIs.

## Discussion

### Summary of findings

Ninety-three percent of health policy and services journals had a COI policy. A minority of policies described how the disclosed COIs of authors would impact the editorial process; none had clear-cut criteria for rejection based on the content of the disclosure. About a fifth of policies explicitly stated that inaccurate or incomplete disclosures might lead to manuscript rejection or retraction. No policy described whether the journal would verify the accuracy or completeness of authors’ disclosed COIs. For financial COIs, the majority asked for specification of the source of payment; a minority asked for the amount (Table 3). Conversely, 81% of policies explicitly required disclosure of non-financial COIs (Table 2).

### Strengths and limitations

This is the first study to investigate the COI disclosure requirements in health policy and services journals, and only the second study investigating how health journals address the potential impact of COI disclosures and incomplete or inaccurate COI disclosures on the editorial process [18]. We used systematic methods for data abstraction (i.e. in duplicate), and evaluated the actual implementation of the COI disclosure policy during the submission process. We do not believe that any of the authors had biases against any of the included journals. Specifically, the team members who abstracted data were naïve to the list of included journals and had no personal experience with any of them. One limitation of this study is our inability to capture the COI policy details that might emerge during the later stages of the editorial process. Another limitation of our study is that we included journals listed under the category of ‘Health Policy and Services’ by the Web of Science. This list might not have included journals listed under similar categories in other databases such as PubMed. However, we do not have a reason to suspect that this might have affected the representativeness of our sample.

### Comparison to similar studies

We have recently conducted a very similar survey addressing policies for COI disclosure but focused on the core clinical journals. All but one of the 117 clinical journals (99%) had a policy for the disclosure of COIs [18], compared to 93% of health policy journals. Two other studies found that 87% of oncology journals and 95% of high-impact biomedical journals adopted COI policies [19, 20].

Eighty-one percent of health policy journals’ policies asked authors to disclose their non-financial COIs, a percentage higher than what previous studies have found. Shawwa et al. [18] recently found that 57% of the core clinical journals asked authors to disclose at least one form of non-financial COI. Earlier, Kesselheim et al. [20] found that 42% of oncology journals asked for non-financial COI disclosure. Health policy journals seem to appreciate the potential impact of non-financial COIs in this field, as compared to other fields (e.g. clinical) where the focus has been on financial COIs.

Table 5 compares the findings of this study to those of a similar study focused on clinical journals [18]. Similar percentages of health policy and clinical journals mentioned a potential impact of inaccurate or incomplete disclosure of COIs on the editorial process (21% and 23%, respectively) [18]. However, medical journals described more severe implications than health policy journals, including manuscript rejection or retraction, ‘sanctions’ and prohibition from future submissions. Moreover, health policy journals were less likely than medical journals to report verifying the disclosed COI (0% vs. 17%).

**Table 5:** Comparison of the requirements of health policy and services journals to those of clinical journals

Variable	Health Policy and Services Journals (n = 72)	Clinical Journals [18] (n = 117)
COI disclosure	67/72 (93)	116/117 (99)
Disclosure policy specifies financial COI	62/67 (93)	116/116 (100)
Disclosure policy specifies non-financial COI	54/67 (81)	66/116 (57)
Impact of authors’ COI disclosure statements on the editorial process	23/67 (34)	20/116 (17)
Impact of inaccurate of incomplete disclosures statements	14/67 (21)	27/116 (23)
Verification the accuracy or completeness of authors’ disclosed COIs	0/67 (0)	20/116 (17)

*COI* conflicts of interest

### Implications for editorial processes

Box 1 provides suggestions for items that journals could include in their policies for authors’ disclosure of COIs. Indeed, the editor of a German journal reported that the introduction in 2005 of a policy requiring all authors to disclose their COI increased disclosures from none in 2002 to 30% of the published articles in 2006 [21]. Given our findings, health policy journals could improve on specific items such as definitions and instructions for disclosing non-financial COIs.

**Box 1:** Suggestions for items that journals could include in their policies for authors’ disclosure of conflicts of interest (COI)

Describe the COI
Specify the level of COI – individual vs. institutional
Specify the type of COI – financial vs. non- financial
If financial COI, specify: • The subtype (e.g. grant; serving as an advisor, consultant or public advocate; stock ownership; indirect financial support (examples include paid by the entity, writing assistance, administrative support); personal fees; direct employment; honoraria for speaking, writing or reviewing on the topic discussed in the manuscript; speaker bureaus or board membership; royalties; patents) • The monetary value • Date • Source and source type (e.g. private for-profit, private not-for-profit, governmental, institution, medical professional society, inter-governmental)
If non-financial COI, specify: • The subtype (e.g. participation in guideline panel, public expression of opinion, religious beliefs, political affiliation) • Date

Although there is little evidence regarding the amount of money that is likely to bias decisions and judgments, it may nevertheless be advisable for journals to ask for more specific details about financial COIs, including the amount of money involved. Lewison et al. [22] suggested a COI statements registry, where authors could declare their competing interests, that could be effortlessly followed over time to verify the authors’ declared interest. Requirement for reviewers and the editorial team to disclose their own COIs, and how these are managed, represents another major area where policies could be improved (e.g. editors withdrawing from handling manuscripts on which they could be conflicted).

### Implications for research

Further insights into conflicts of interest issues will require a framework that provides clear typology and operational definitions to facilitate both the disclosure of COIs by authors and reviewers and their assessment and management by the editorial team. Optimal progress will also necessitate development of a valid and reliable approach to verify the different types of disclosed COI. Future research should also build the evidence base for the different interventions and policies intended to manage COIs.

## Conclusion

The objective of this study was to assess the policies of health policy and services journals for authors to disclose financial and non-financial COIs. While the majority of the policies required the disclosure of financial and non-financial COIs of authors, few required details on disclosed COIs. We also found that a small minority of policies specified how the disclosed COIs would impact the editorial process or required COI disclosure for reviewers and editors. This may jeopardise the published evidence to bias, which may be reflected on the health policies. Our findings should assist journals in improving their COI disclosure policies. Detailed COI disclosures will help researchers and policymakers in building unbiased evidence-informed decisions taking into account the possibilities of competing interests imposed on their policy plans.

## Additional files

Additional file 1: Potential impact of disclosed COIs on the editorial process. (DOCX 14 kb)

Additional file 2: Potential impact of inaccurate or incomplete disclosure of COIs on the editorial process. (DOCX 13 kb)

## Abbreviations

COI: Conflicts of interest
COPE: Committee on Publication Ethics
FCTC: Framework Convention on Tobacco Control
ICMJE: International Committee of Medical Journal Editors
