# Supplementary material for: Requirements of health policy and services journals for authors to disclose financial and non-financial conflicts of interest: a cross-sectional study
Source: Health Res Policy Syst. 2017 Sep 19;15:80. doi: 10.1186/s12961-017-0244-2 (PMC5606121; doi:10.1186/s12961-017-0244-2)
Supplement: Supplementary file 1 — Potential impact of disclosed COIs on the editorial process. (DOCX 14 kb) [file 12961_2017_244_MOESM1_ESM.docx]

**Additional file** **1.** Potential impact of disclosed COIs on the editorial process

| Journal | Comment |
| --- | --- |
| BMJ Quality & Safety | 'We will not reject papers simply because authors have a competing interest, but these will be declared on the published paper.' |
| Value In Health | The journal may decide not to publish on the basis of declared conflict. |
| Health Services Research* | Website note:  Disclosure process: At HSR we emphasize disclosure of sponsors and relationships to related advocacy groups to the editors and reasonable publication of such information. With the exception of 'review and approve' clauses that raise the possibility of censorship, such information will not enter into our decisions to accept or reject a manuscript.  Submission note:  Conflicts of interest would rarely preclude the publication of an article in HSR, if disclosed fully. Knowing in advance about conflicts, whether financial or not, whether real or perceived, will allow us to select reviewers and handle the manuscript appropriately. Please check 'yes' if there are any conflict of interest issues and then discuss them in your cover note.  Disclosure form: We anticipate that the vast majority of 'conflicts,' if disclosed in advance, will have little bearing on a reader's assessment of the research and no bearing on our decision to publish. |
| Medical Care Research And Review | Articles will be evaluated fairly and will not necessarily be rejected when any competing interests are declared. |
| Pharmacoeconomics | ‘We will not reject manuscripts simply because the authors have a conflict of interest, but we will publish a declaration in the manuscript as to whether or not the authors have conflicts of interests.’ |
| Journal Of Health Economics | The journal may decide not to publish on the basis of declared conflict. |
| Psychiatric Services | If it appears that there may be, then further review and possible rejection of the manuscript may occur. |
| Patient-Patient Centered Outcomes Research | ‘We will not reject manuscripts simply because the authors have a conflict of interest, but we will publish a declaration in the manuscript as to whether or not the authors have conflicts of interests.’ |
| Evaluation & The Health Professions | Articles will be evaluated fairly and will not necessarily be rejected when any competing interests are declared. |
| Health Policy | The journal may decide not to publish on the basis of declared conflict. |
| AIDS Care-Psychological And Socio-Medical Aspects Of AIDS/HIV | The journal editor will use this information to inform his or her editorial decisions, and may publish such disclosures to assist readers in evaluating the article. The editor may decide not to publish your article on the basis of any declared conflict. |
| Journal Of Health Services Research & Policy | Any commercial or financial involvements that might present an appearance of a conflict of interest related to the Contribution are disclosed in a covering letter accompanying the Contribution and all such potential conflicts of interest will be discussed with the Editor as to whether disclosure of this information with the published Contribution is to be made in the journal. Articles will be evaluated fairly and will not necessarily be rejected when any competing interests are declared. |
| Journal Of Aging And Health | Any commercial or financial involvements that might present an appearance of a conflict of interest related to the Contribution are disclosed in a covering letter accompanying the Contribution and all such potential conflicts of interest will be discussed with the Editor as to whether disclosure of this information with the published Contribution is to be made in the journal. Articles will be evaluated fairly and will not necessarily be rejected when any competing interests are declared. |
| Journal Of Interprofessional Care | The journal editor will use this information to inform his or her editorial decisions, and may publish such disclosures to assist readers in evaluating the article. The editor may decide not to publish your article on the basis of any declared conflict. |
| Journal Of Pediatric Health Care | The journal may decide not to publish on the basis of declared conflict. |
| Qualitative Health Research | Articles will be evaluated fairly and will not necessarily be rejected when any competing interests are declared. |
| Disability And Health Journal | The journal may decide not to publish on the basis of declared conflict. |
| International Journal Of Health Planning And Management* | Papers will not be rejected because there is a competing interest, but a declaration on whether or not there are competing interests will be added to the paper.  The journal editor, Editorial Board members and other editorial staff (including peer reviewers) withdraw from discussions about submissions where any circumstances might prevent him/her offering unbiased editorial decisions. When the editor is presented with a paper where his own interests may impair his ability to make an unbiased editorial decision, he deputises decisions about the paper to a suitably qualified individual. |
| Health Information Management Journal | Articles will be evaluated fairly and will not necessarily be rejected when any competing interests are declared. Any commercial or financial involvements that might present an appearance of a conflict of interest related to the Contribution are disclosed in a covering letter accompanying the Contribution and all such potential conflicts of interest will be discussed with the Editor as to whether disclosure of this information with the published Contribution is to be made in the journal. |
| International Journal Of Health Services | Any commercial or financial involvements that might present an appearance of a conflict of interest related to the Contribution are disclosed in a covering letter accompanying the Contribution and all such potential conflicts of interest will be discussed with the Editor as to whether disclosure of this information with the published Contribution is to be made in the journal. Articles will be evaluated fairly and will not necessarily be rejected when any competing interests are declared. |
| Eastern Mediterranean Health Journal | EMHJ will not reject a paper solely on the basis of a declared competing interest but will take this into consideration when assessing a paper and, if considered relevant, it may be included in the published paper. |
| Health Sociology Review | The journal editor will use this information to inform his or her editorial decisions, and may publish such disclosures to assist readers in evaluating the article. The editor may decide not to publish your article on the basis of any declared conflict. |
| Inquiry-The Journal Of Health Care Organization Provision And Financing | Articles will be evaluated fairly and will not necessarily be rejected when any competing interests are declared. Any commercial or financial involvements that might present an appearance of a conflict of interest related to the Contribution are disclosed in a covering letter accompanying the Contribution and all such potential conflicts of interest will be discussed with the Editor as to whether disclosure of this information with the published Contribution is to be made in the journal. |

*Statement interpreted as COIs would have no effect on the decision to accept or reject the paper.
