# Supplementary material for: Requirements of health policy and services journals for authors to disclose financial and non-financial conflicts of interest: a cross-sectional study
Source: Health Res Policy Syst. 2017 Sep 19;15:80. doi: 10.1186/s12961-017-0244-2 (PMC5606121; doi:10.1186/s12961-017-0244-2)
Supplement: Supplementary file 2 — Potential impact of inaccurate or incomplete disclosure of COIs on the editorial process. (DOCX 13 kb) [file 12961_2017_244_MOESM2_ESM.docx]

**Additional** **file** **2.** Potential impact of inaccurate or incomplete disclosure on the editorial process

| Journal | Comment |
| --- | --- |
| Pharmacoeconomics | The Editors reserve the right to reject manuscripts that do not comply with the above-mentioned guidelines. The author will be held responsible for false statements or failure to fulfill the above-mentioned guidelines. |
| Quality Of Life Research | The Editors reserve the right to reject manuscripts that do not comply with the above-mentioned guidelines. The author will be held responsible for false statements or failure to fulfill the above-mentioned guidelines. |
| Administration And Policy In Mental Health And Mental Health Services Research | The Editors reserve the right to reject manuscripts that do not comply with the above-mentioned guidelines. The author will be held responsible for false statements or failure to fulfill the above-mentioned guidelines. |
| European Journal Of Health Economics | The Editors reserve the right to reject manuscripts that do not comply with the above-mentioned guidelines. The author will be held responsible for false statements or failure to fulfill the above-mentioned guidelines. |
| Patient-Patient Centered Outcomes Research | The Editors reserve the right to reject manuscripts that do not comply with the above-mentioned guidelines. The author will be held responsible for false statements or failure to fulfill the above-mentioned guidelines. |
| AIDS Care-Psychological And Socio-Medical Aspects Of AIDS/HIV | If a conflict of interest is not declared to the journal upon submission, or during review, and it affects the actual or potential interpretation of the results, the paper may be rejected or retracted. |
| Journal Of Interprofessional Care | If a conflict of interest is not declared to the journal upon submission, or during review, and it affects the actual or potential interpretation of the results, the paper may be rejected or retracted. |
| Journal Of Community Health | The Editors reserve the right to reject manuscripts that do not comply with the above-mentioned guidelines. The author will be held responsible for false statements or failure to fulfill the above-mentioned guidelines. |
| Health Care Management Science | The Editors reserve the right to reject manuscripts that do not comply with the above-mentioned guidelines. The author will be held responsible for false statements or failure to fulfill the above-mentioned guidelines. |
| Disability And Health Journal | Omission of relevant information may lead to rejection of the submission at any stage in the process. |
| Community Mental Health Journal | The Editors reserve the right to reject manuscripts that do not comply with the above-mentioned guidelines. The author will be held responsible for false statements or failure to fulfill the above-mentioned guidelines. |
| Health Care Analysis | The Editors reserve the right to reject manuscripts that do not comply with the above-mentioned guidelines. The author will be held responsible for false statements or failure to fulfill the above-mentioned guidelines. |
| Health Sociology Review | If a conflict of interest is not declared to the journal upon submission, or during review, and it affects the actual or potential interpretation of the results, the paper may be rejected or retracted. |
| International Journal Of Health Economics And Management | The Editors reserve the right to reject manuscripts that do not comply with the above-mentioned guidelines. The author will be held responsible for false statements or failure to fulfill the above-mentioned guidelines. |
